# JNK and Autophagy Independently Contributed to Cytotoxicity of Arsenite combined With Tetrandrine *via* Modulating Cell Cycle Progression in Human Breast Cancer Cells

**DOI:** 10.3389/fphar.2020.01087

**Published:** 2020-07-17

**Authors:** Bowen Yu, Bo Yuan, JingZhe Li, Anna Kiyomi, Hidetomo Kikuchi, Hideki Hayashi, Xiaomei Hu, Mari Okazaki, Munetoshi Sugiura, Toshihiko Hirano, Yingyi Fan, Xiaohua Pei, Norio Takagi

**Affiliations:** ^1^ Department of Applied Biochemistry, School of Pharmacy, Tokyo University of Pharmacy & Life Sciences, Tokyo, Japan; ^2^ Galactophore Department, Beijing University of Chinese Medicine Third Affiliated Hospital, Beijing, China; ^3^ Laboratory of Pharmacology, School of Pharmacy, Josai University, Saitama, Japan; ^4^ Beijing Key Laboratory of Research of Chinese Medicine on Prevention and Treatment for Major Diseases, Experimental Research Center, China Academy of Chinese Medical Sciences, Beijing, China; ^5^ Drug Safety and Risk Management, School of Pharmacy, Tokyo University of Pharmacy & Life Sciences, Tokyo, Japan; ^6^ Laboratory of Pharmacotherapy, Department of Clinical Dietetics and Human Nutrition, Faculty of Pharmacy and Pharmaceutical Sciences, Josai University, Saitama, Japan; ^7^ Hematology Department, XiYuan Hospital, China Academy of Chinese Medical Sciences, Beijing, China; ^8^ Clinical Pharmacology, School of Pharmacy, Tokyo University of Pharmacy & Life Sciences, Tokyo, Japan

**Keywords:** arsenite, tetrandrine, breast cancer cells, JNK, combination therapy, cell cycle arrest, autophagy

## Abstract

Novel therapeutic strategies for breast cancer are urgently needed due to the sustained development of drug resistance and tumor recurrence. Trivalent arsenic derivative (arsenite, As^III^) has been reported to induce cytotoxicity in breast cancer cells. We recently demonstrated that As^III^ plus tetrandrine (Tetra), a Chinese plant-derived alkaloid, exerted potent antitumor activity against human breast cancer cells, however, the underlying mechanisms for their action have not been well defined. In order to provide fundamental insights for understanding the action of As^III^ plus Tetra, the effects of the combined regimen on two breast cancer cell lines T47D and MDA-MB-231 were evaluated. Compared to T47D cells, MDA-MB-231 cells were much more susceptible to the synergistic cytotoxic effects of As^III^ and Tetra. Besides the induction of apoptotic/necrotic cell death, S-phase arrest and autophagic cell death were also observed in MDA-MB-231 cells. Exposure of MDA-MB-231 cells to As^III^ and Tetra caused the activation of MAPKs. Cytotoxicity of the combined regimen in MDA-MB-231 cell was significantly abrogated by SP600125, a potent c-Jun N-terminal kinase (JNK) inhibitor. However, similar abrogation was not caused by p38 and ERK inhibitors. The addition of either autophagy inhibitors (3-methyladenine or wortmannin) or SP600125 corrected the combined regimen-triggered S-phase arrest, whereas had little effect on the apoptosis/necrosis induction in the cells. Surprisingly, SP600125NC, a negative control for SP600125, significantly strengthened S-phase arrest and the cytotoxicity induced by the combined regimen. The addition of SP600125 did not alter autophagy induction. In conclusion, the cytotoxicity of As^III^ combined with Tetra was attributed to the induction of S-phase arrest, apoptotic/necrotic and autophagic cell death. The enhanced cytotoxicity of the two drugs by SP600125NC might be explained by its capability to strengthen S-phase arrest. Our results suggested that JNK and autophagy independently contributed to the cytotoxicity *via* modulating cell cycle progression. The study further provides fundamental insights for the development of As^III^ in combination with Tetra for patients with different types of breast cancer.

## Introduction

In spite of recent progress in early detection, diagnosis, and targeted treatment options, breast cancer is still the most frequently diagnosed cancer among women worldwide and one of the leading causes of cancer-related deaths for women (Taylor et al., 2015; Fitzmaurice et al., 2017). Novel therapeutic strategies are urgently needed due to the sustained development of drug resistance, tumor recurrence, and metastasis (Taylor et al., 2015; Fitzmaurice et al., 2017).

It has been demonstrated that arsenic trioxide (As_2_O_3_, a trivalent arsenic derivative) exhibits high therapeutic efficacy against relapsed and refractory acute promyelocytic leukemia patients. The great therapeutic achievements thus encouraged more researchers to explore its potential future application for other malignant neoplasms (Dilda and Hogg, 2007; Yuan et al., 2010). We have demonstrated that arsenic compounds such as arsenic disulfide (As_2_S_2_) exhibits inhibitory effects against various types of cancer cells including breast cancer cell lines (Hu et al., 2014a; Hu et al., 2014b; Zhao et al., 2018a; Zhao et al., 2018b; Zhao et al., 2019). We also demonstrated the differentiation-inducing activity of clinically achievable concentrations of arsenite (As^III^, a trivalent arsenic compound) combined with tetrandrine (Tetra), a traditional Chinese herbal medicine, in breast cancer cell lines (Yu et al., 2019). We thus suggested that the combined regimen of As^III^ and Tetra should be valuable in the development of differentiated therapeutic approach to combat breast cancer. In addition, we demonstrated cytocidal effect of As^III^ against estrogen receptor (ER)-positive MCF-7 breast cancer cell line, and indicated that Tetra synergistically strengthened the cytotoxicity of As^III^ (Yao et al., 2017). Our recent *in vitro* and *in vivo* study also demonstrated antitumor activity of As^III^ combined with Tetra against human triple-negative breast cancer (TNBC) cell line MDA−MB−231 (Yuan et al., 2018).

Anti-cancer therapy involves many novel therapeutic interventions, such as modification of tumor microenvironment, innate immune gene response, the induction of apoptotic and/or autophagic cell death in premalignant and malignant cells (Yao et al., 2017; Yoshino et al., 2018; Khare et al., 2019). Additionally, the role of necrotic cell death in chemotherapeutic treatment has been increasing appreciated since tumor cells evolve diverse strategies to evade apoptosis during tumor development (Cui et al., 2011; Xu et al., 2014). In this regard, we have demonstrated that autophagic and necrotic cell death contributed to the cytocidal effects of As^III^ in combination with Tetra in breast cancer cells (Yuan et al., 2018). In addition, S-phase arrest associated with the alterations of cell cycle regulators such as p21, p27 and cyclin D1 was also observed (Yuan et al., 2018). Despite this, the correlation between S-phase arrest and autophagic/necrotic cell death has not yet been clarified.

Mitogen-activated protein kinases (MAPKs) are known to be involved in a variety of cellular responses including cell division, proliferation, differentiation and cell death. The MAPKs include c-Jun NH2-terminal protein kinase (JNK), p38 kinase and extracellular signal-regulated kinase (ERK) (Cargnello and Roux, 2011). ERK usually serves as a survival mediator implicated in cytoprotection (Kikuchi et al., 2013; Kawiak et al., 2019). On the other hand, JNK and p38 MAPK are generally considered to be involved in cell death induction by diverse stimuli (Hu et al., 2014b; Kikuchi et al., 2014; Deng et al., 2018; Qiao et al., 2019). Of note, recent emerging evidence has demonstrated a strong association between the activation of JNK and antitumor agent-mediated cytotoxicity such as cell cycle arrest as well as autophagic cell death in breast cancer cells (Wang et al., 2016; Xie et al., 2017; Kong et al., 2020). Our previous report has demonstrated the contribution of S-phase arrest, autophagic and necrotic cell death to the cytotoxicity of As^III^ combined with Tetra in breast cancer cell line MDA-MB-231 (Yuan et al., 2018). However, whether the activation of MAPKs occurs and links to the combined regimen-triggered cellular responses have not yet been investigated.

A previous study (Yu et al., 2017) has demonstrated a clear difference between MCF-7 and T47D cells in the response to progesterone, although both MCF-7 and T47D are ER-positive breast cancer cell lines and share the similarities in phenotypic and molecular characteristics (Aka and Lin, 2012). In this study, in order to provide fundamental insights for understanding the action of As^III^ combined with Tetra in breast cancer cells, the cytotoxicity of the combined regimen was first evaluated in both T47D and MDA-MB-231 cells. The relation between autophagic cell death and apoptotic/necrotic cell death as well as cell cycle arrest was also explored in MDA-MB-231 cells, which showed a relatively high susceptibility to the combined regimen. Given critical roles of MAPKs in a variety of cellular responses, the relation between its activation and the combined regimen-mediated cytotoxicity was also evaluated. The association of activation of JNK, which was found to be closely related to the cytotoxicity, with various cellular responses such as cell cycle progression and autophagic cell death was further clarified. Tamoxifen (TAM) is known as a selective ER modulator and has been widely used in chemotherapy of breast cancer. Since previous reports have demonstrated that TAM induced cytotoxicity including apoptosis in different types of breast cancer cells regardless of ER status (Liu et al., 2014; Yeh et al., 2014), TAM was used as a positive control in the current study.

## Materials and Methods

### Materials

Sodium arsenite (NaAsO_2_, As^III^) (>99% purity) and tetrandrine (99.2% purity) were purchased from Tri Chemical Laboratories (Yamanashi, Japan) and National Institutes for Food and Drug Control (Beijing, China), respectively. Fetal bovine serum (FBS) was purchased from Nichirei Biosciences (Tokyo, Japan). Dulbecco’s modified Eagle’s medium (DMEM), RPMI-1640 medium, phenazine methosulfate (PMS), and dimethyl sulfoxide (DMSO) were obtained from Wako Pure Chemical Industries (Osaka, Japan). Propidium iodide (PI),　proteinase K, ribonuclease A (RNaseA), 2,3-bis(2-methoxy-4-nitro-5-sulfophenyl)3.2-5-[(phenylamino)carbony]-2*H*-tetrazolium hydroxide (XTT), and tamoxifen were purchased from Sigma-Aldrich (St. Louis, MO, USA). MAPK inhibitors and their negative controls (JNK inhibitor SP600125 and its negative control SP600125NC; p38 MAPK inhibitor SB203580 and its negative control SB202474; ERK inhibitor PD98059) were purchased from Calbiochem (La Jolla, CA, USA). Autophagy inhibitors, 3-methyladenine (3-MA) and wortmannin, were purchased from Wako Pure Chemical Industries and Calbiochem, respectively. Can Get Signal^®^ Immunoreaction Enhancer Solution was purchased from Toyobo Co., Ltd. (Osaka, Japan).

### Cell Culture and Treatment

Breast cancer cell lines, MDA-MB-231 and T47D, were obtained from the American Type Culture Collection (ATCC, Manassas, VA, USA) and the Health Science Research Resources Bank (HSRRB, Osaka, Japan), respectively. MDA-MB-231 cells were cultured in DMEM medium, and T47D cells were cultured in RPMI-1640 medium, both of which were supplemented with 10% heat-inactivated FBS and 100 U/ml of penicillin and 100 µg/ml of streptomycin, in a humidified 5% CO_2_ atmosphere at 37°C. Based on our recent work (Yuan et al., 2018), both cancer cells were treated with various concentrations of As^III^ (5, 10, and 15 μM) and Tetra (5.6, 6.4, and 7.2 μM), alone or in combination, for 48 h. Tetra was dissolved in DMSO, and no cytotoxicity of the final concentrations of DMSO was observed in the current experimental system.

### Cell Viability Assay

The cell viability was measured by XTT dye-reduction assay as described previously (Yoshino et al., 2018; Yuan et al., 2019). Relative cell viability was expressed as the ratio of the absorbance of each treatment group against that of the corresponding untreated control group. Data are shown as mean ± standard deviation (SD) from more than three independent experiments. In order to evaluate whether the two drugs, As^III^ and Tetra, generated synergistic, antagonistic, or additive effects, a combination index (CI) was determined as reported previously, using the computer software ComboSyn (Combosyn Inc. NJ, USA) for drug combinations and for general dose–effect analysis, which was developed by Chou (2006; 2010). The effect of the combination treatment was defined as a synergistic effect if CI < 1, an additive effect if CI = 1 or an antagonistic effect if CI > 1 (Chen et al., 2014; Yao et al., 2017). In order to evaluate whether the activation of JNK, p38 and ERK is implicated in the cytotoxicity of As^III^ and Tetra against MDA-MB-231 cells, which possessed a relatively high susceptibility to the combinational treatment in the current study, the cells were treated with respective potent inhibitor at the indicated concentrations for 30 min prior to treatment with 10 μM As^III^+6.4 μM Tetra in the presence or absence of each inhibitor for an additional 48 h, followed by the XTT assay as described above.

### Annexin V/PI Analysis

The TACS™ Annexin V-FITC apoptosis detection Kits (Trevigen, MD, USA) was used for the detection of apoptotic and necrotic cells according to the method described previously (Yuan et al., 2015; Yoshino et al., 2018). Briefly, after treatment for 48 h with various concentrations of As^III^ (5, 10, and 15 μM) and Tetra (5.6, 6.4, and 7.2 μM), alone or in combination, cells were washed with PBS. Cells were then incubated for 15 min in 100 μl of reaction buffer, which containing annexin V-FITC and PI, followed by addition of 400 μl of binding buffer. Fluorescence intensities of FITC and PI were measured by a FACSCanto flow cytometer (Becton Dickinson, San Jose, CA, USA). A total of 30,000 events were acquired and data were analyzed by Diva software. Annexin V(−)PI(−), annexin V(+)PI(−), annexin V(+)PI(+), and annexin V(−)PI(+) cells were defined as viable, early apoptotic, late apoptotic, and necrotic cells, respectively. In order to clarify whether autophagy induction or the activation of JNK is associated with the induction of apoptosis and necrosis, MDA-MB-231 cells were treated with either autophagy inhibitors (3-MA or wortmannin) or JNK inhibitor (SP600125), at the indicated concentrations for 30 min prior to treatment with 10 μM As^III^+6.4 μM Tetra in the presence or absence of each inhibitor for an additional 48 h, followed by the annexin V/PI as described above.

### Cell Cycle Analysis

After treatment with 10 μM As^III^+6.4 μM Tetra for 48 h, cell cycle analysis was performed using a FACSCanto flow cytometer (Becton–Dickinson) according to a method reported previously (Kikuchi et al., 2013; Yao et al., 2017). Briefly, cells were washed twice with PBS, fixed with 1% paraformaldehyde/PBS for 30 min, washed twice again with PBS, permeabilized in 70% (v/v) cold ethanol and kept at −20°C for at least 4 h. Cell pellets were then washed twice with PBS after centrifugation and incubated with 0.25% Triton-X 100 for 5 min on ice. After centrifugation and washing with PBS, cells were resuspended in 500 μl of PI/RNase A/PBS (5 μg/ml of PI and 0.1% RNase A in PBS) and incubated for 30 min in the dark at room temperature. A total of 10,000 events were acquired and Diva software and Mod-Fit LT™ Ver.3.0 (Verity Software House, ME, USA) were used to calculate the number of cells at each G_0_/G_1_, S and G_2_/M phase fraction. In order to explore whether JNK or autophagy contributes to the cytotoxicity of the combined regimen by modulating cell cycle progression, MDA-MB-231 cells were treated with JNK inhibitor or autophagy inhibitors at the indicated concentrations for 30 min prior to treatment with 10 μM As^III^+6.4 μM Tetra in the presence or absence of each inhibitor for an additional 48 h, followed by the cell cycle analysis as described above.

### Western Blot Analysis

For protein samples preparation, cell pellets (approximately 1-2×10^6^ cells per 110 μl Laemmli buffer) were suspended in lysis buffer (Laemmli buffer containing 100 mM DTT, 2 μg/ml leupeptin, 2 μg/ml aprotinin, 1 μg/ml pepestain, 1 mM PMSF). The suspensions of cells were sonicated using a sonicator (Qsonica, LLC, CT, USA) with 10 short bursts of 2 s followed by intervals of 2 s for cooling. The suspensions were kept at all times in an ice bath. Sonicated cells were heated in 95°C for 5 min, and then centrifuged at 13,000 g for 15 min at 4°C. Protein concentrations of the supernatant were determined according to Bradford’s method using the protein assay dye reagent (Bio-Rad, CA, USA) according to the manufacturer’s instructions, and using BSA as the standard. Western blot analysis was carried out according to the methods previously described (Yuan et al., 2009). Briefly, after separation of proteins on a sodium dodecyl sulfate (SDS) polyacrylamide gel electrophoresis, followed by transferring to a polyvinylidene difluoride (PVDF) membrane (Millipore Corp, MA, USA), protein bands were detected using the following primary antibodies and dilution ratios: mouse anti-human β-actin (1:5,000 dilution; cat. no. A-5441; Sigma–Aldrich, MO, USA), rabbit anti-human LC3 (1:1,000 dilution; cat. no. 12741), rabbit anti-human phospho-SAPK/JNK (Thr183/Tyr185, 1:1,000 dilution; cat. no. 9251) and SAPK/JNK (1:1,000 dilution; cat. no. 9252), rabbit anti-human phospho-p38 (Thr180/Tyr182, 1:1,000 dilution; cat. no. 9211) and p38 (1:1,000 dilution; cat. no. 9212), rabbit anti-human phospho-p44/42 MAPK (Erk1/2) (Thr202/Tyr204) (1:2000 dilution; cat. no. 4370) and p44/42 MAPK (Erk1/2) (137F5) (1:1,000 dilution; cat. no. 4695) (Cell Signaling Technology, MA, USA). Blotted protein bands were detected with respective horseradish peroxidase-conjugated secondary antibody and an enhanced chemiluminescence (ECL) Western blot analysis system (Amersham Pharmacia Biotech, Buckinghamshire, UK). Relative amounts of the immunoreactive proteins were calculated from the density of the gray level on a digitized image using a program, NIH Image 1.60.

### Statistical Analysis

Experiments were independently repeated three times, and the results are presented as the means ± SD of the three assays. Statistical analysis was conducted using one-way ANOVA followed by Dunnett’s post-test. A probability level of p<0.05 was considered to indicate a statistically significant difference.

## Results

### Synergistic Cytotoxicity of As^III^ Combined With Tetra in Human Breast Cancer Cell Lines

**Figure 1:**
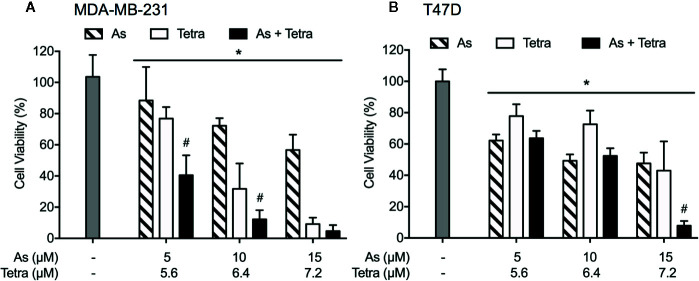
Synergistic cytotoxicity of As^III^ combined with Tetra in human breast cancer cell lines. Following treatment with various concentrations of As^III^ alone (5, 10, and 15 µM), Tetra alone (5.6, 6.4, and 7.2 μM), or their combination for 48 h, the cell viability of MDA-MB-231 **(A)** and T47D **(B)** was determined by XTT assay. Relative cell viability was calculated as the ratio of the absorbance at 450 nm of each treatment group against those of the corresponding untreated control group. Data are shown as the means and SD from more than three independent experiments. *p<0.05 vs. control; ^#^p<0.001 vs. each alone. As, As^III^; Tetra, tetrandrine.

Cell viability of MDA-MB-231 and T47D cells was determined by XTT assay following treatment for 48 h with As^III^ and Tetra, alone or in combination, at the indicated concentrations. As shown in **Figure 1**, treatment with As^III^ alone (5, 10, and 15 μM) resulted in a similar growth inhibition in both cancer cells. Tetra alone (5.6, 6.4, and 7.2 μM) induced a clear dose-dependent decrease in cell viability of MDA-MB-231, but not in T47D, indicating that MDA-MB-231 cells were more sensitive to Tetra compared to T47D. Intriguingly, synergistic cytocidal effect of the two drugs was observed in MDA-MB-231 cells when treated with a combination of a relatively low concentration of As^III^ and Tetra (CI values were 0.9951 and 0.9967 for the treatment of 5 μM As^III^+5.6 μM Tetra and 10 μM As^III^+6.4 μM Tetra, respectively). However, similar synergistic effect was only observed in T47D cells following the treatment with a combination of a relatively high concentration of the two drugs (CI value was 0.6921 for the treatment of 15 μM As^III^+7.2 μM Tetra). These results thus indicated that the sensitivity of MDA-MB-231 to the combinatorial treatment was substantially higher than that of T47D.

### Contribution of Apoptosis and Necrosis to the Cytotoxicity of As^III^ Combined With Tetra in Breast Cancer Cells

**Figure 2:**
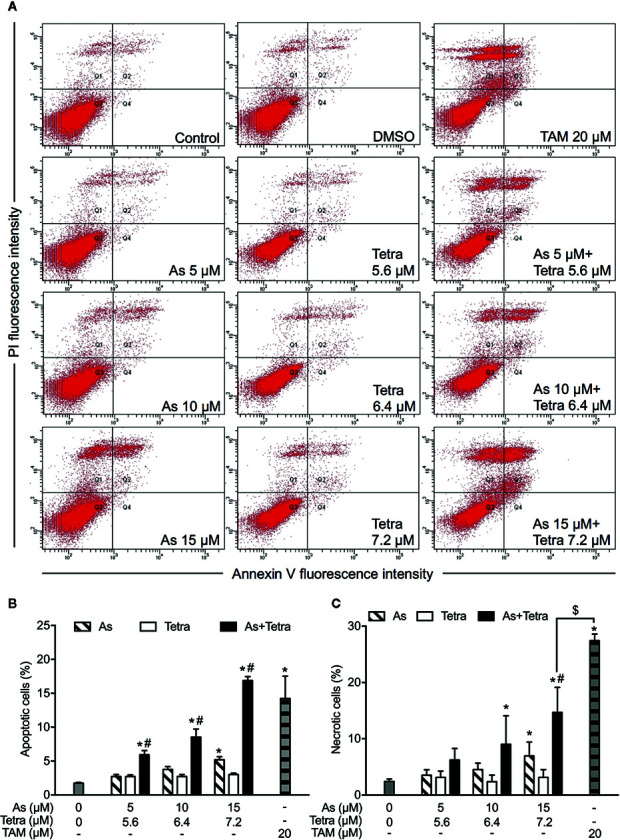
Induction of apoptotic and necrotic cell death in MDA-MB-231 cells by As^III^ combined with Tetra. **(A)** After treatment with indicated concentrations of As^III^ and Tetra, alone or in combination, for 48 h, cells were stained with annexin V-FITC and PI, and analyzed by flow cytometry. Annexin V(−)PI(−) cells, annexin V(+)PI(−) cells, annexin V(+)PI(+) cells, and annexin V(−)PI(+) cell represent viable cells, early apoptotic cells, late apoptotic/necrotic cells, and necrotic cells, respectively. **(A)** Representative dot plots from three independent experiments are shown. Quantifications in the percentages of apoptotic cells **(B)** and necrotic cells **(C)** are shown, respectively. *p<0.05 vs. control; ^#^p<0.05 vs. each alone; ^$^p<0.01 vs. TAM. TAM (20 µM) used as an inducer (positive control) for the induction of apoptosis and necrosis. As, As^III^; Tetra, tetrandrine; TAM, tamoxifen.

**Figure 3:**
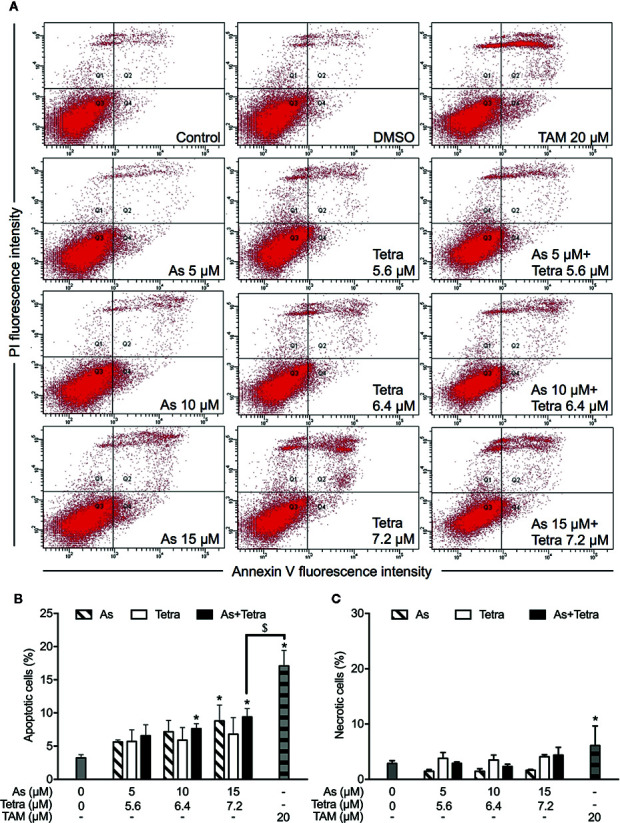
Induction of apoptotic and necrotic cell death in T47D cells by As^III^ combined with Tetra. **(A)** After treatment with indicated concentrations of As^III^ and Tetra, alone or in combination, for 48 h, cells were stained with annexin V-FITC and PI, and analyzed by flow cytometry. Annexin V(−)PI(−) cells, annexin V(+)PI(−) cells, annexin V(+)PI(+) cells, and annexin V(−)PI(+) cell represent viable cells, early apoptotic cells, late apoptotic/necrotic cells, and necrotic cells, respectively. **(A)** Representative dot plots from three independent experiments are shown. Quantifications in the percentages of apoptotic cells **(B)** and necrotic cells **(C)** are shown, respectively. *p<0.05 vs. control; ^$^p<0.05 vs. TAM. TAM (20 µM) used as an inducer (positive control) for the induction of apoptosis and necrosis. As, As^III^; Tetra, tetrandrine; TAM, tamoxifen.

After exposure of both breast cancer cells for 48 h to the indicated concentrations of As^III^ and Tetra, alone or in combination, annexin V/PI analysis was conducted to explore whether apoptosis and/or necrosis contribute to the cytotoxicity of As^III^ combined with Tetra. TAM, a widely used in chemotherapy of breast cancer, has been shown to induce apoptosis of breast cancer cells including MDA-MB-231 and T47D (Liu et al., 2014; Yeh et al., 2014). In line with these previous reports, both cancer cells treated with 20 μM TAM underwent early and late stage apoptosis in comparison with control group, as evidenced by an increase in the number of annexin V(+)PI(-) and annexin V(+)PI(+) cells (**Figures 2A, B** and **3A, B**). A higher percentage of annexin V(-)PI(+) cells was further detected in MDA-MB-231 compared to T47D cells, indicating the induction of necrotic cell death in both cells by TAM (**Figures 2A, C** and **3A, C**).

In comparison to TAM, treatment with either As^III^ or Tetra alone hardly induced apoptosis, except that the highest concentrations of 15 μM As^III^ slightly but significantly induced apoptosis in both cells (**Figures 2A, B** and **3A, B**). Intriguingly, synergistic apoptosis-inducing activities of As^III^ combined with Tetra, regardless of their concentrations, were observed in MDA-MB-231 cells (CI values were 0.3873, 0.3245, and 0.1204 for the treatment of 5 μM As^III^+5.6 μM Tetra, 10 μM As^III^+6.4 μM Tetra, and 15 μM As^III^+7.2 μM Tetra, respectively) (**Figures 2A, B**). Of note, the combined regimen of 15 μM As^III^+7.2 μM Tetra, and TAM exhibited very similar apoptosis-inducing activity in the cells (**Figures 2A, B**). On the other hand, treatment with the combination of As^III^ and Tetra showed only a modest but significant increase in the apoptosis-inducing activity in T47D cells in comparison with control group (**Figures 3A, B**). Besides apoptosis-inducing activity, necrosis-inducing activity of 15 μM As^III^ was also observed in MDA-MB-231 cells, and further enhanced by the addition of 7.2 μM Tetra (**Figure 2C**). In addition, 10 μM As^III^ combined with 6.4 μM Tetra also exhibited necrosis-inducing activity in the cells (**Figure 2C**). Despite this, no necrosis-inducing activity of the two drugs, either alone or in combination, was recognized in T47D cells (**Figure 3C**). Since a relatively high susceptibility of MDA-MB-231 cells to As^III^ combined with Tetra was observed, a detailed analysis of the cytotoxicity of the combined regimen was carried out using the cells in the following study.

### Autophagy Contributed to the Cytotoxicity of As^III^ Combined With Tetra in MDA-MB-231 Cells by Modulating Cell Cycle Progression

**Figure 4:**
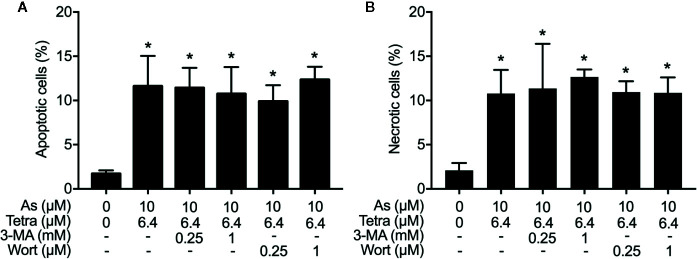
Effects of autophagy inhibitors on the induction of apoptosis and necrosis in MDA-MB-231 cells treated with the combination of As^III^ and Tetra. After treatment with 10 μM As^III^+6.4 μM Tetra in the presence or absence of 3-MA (0.25, 1.0 mM) or wortmannin (0.25, 1.0 μM) for 48 h, cells were stained with annexin V-FITC and PI, and analyzed by flow cytometry. The percentages of apoptotic cells **(A)** and necrotic cells **(B)** were quantified by the same manner as described in the legend of **Figures 2** and **3**. *p<0.0001 vs. control. As, As^III^; Tetra, tetrandrine; Wort, wortmannin.

**Figure 5:**
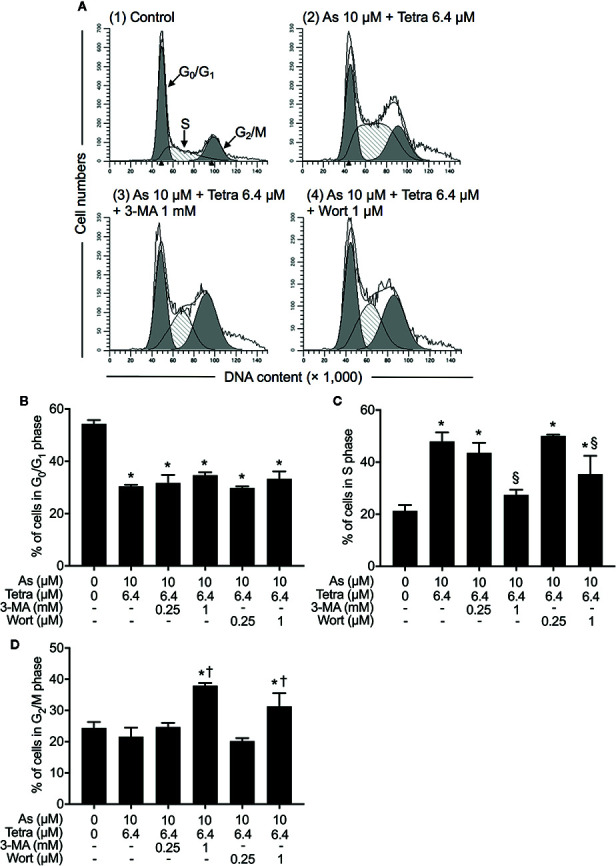
Contribution of autophagy to the cytotoxicity of As^III^ combined with Tetra in MDA-MB-231 cells by modulating cell cycle progression. **(A–D)** After treatment with 10 μM As^III^+6.4 μM Tetra in the presence or absence of 3-MA (0.25, 1.0 mM) or wortmannin (0.25, 1.0 μM) for 48 h, cell cycle profiling was performed by FACSCanto flow cytometer. Analyzed data and profiles for each G_0_/G_1_ and G_2_/M phase using Diva software and ModFit LT™ ver.3.0. are shown in the gray area. Cells at S phase are shown as shaded area. A representative FACS histogram from three separate experiments is shown **(A)**. *p<0.05, vs. control; ^§^p<0.05, ^†^p<0.01, vs. 10 μM As^III^ +Tetra 6.4 μM. As, As^III^; Tetra, tetrandrine; Wort, wortmannin.

We have recently reported the involvement of activation of autophagic cell death in the combined regimen-mediated cytotoxicity of breast cancer cells (Yao et al., 2017; Yuan et al., 2018). We also demonstrated that As^III^ in combination with Tetra induced S-phase arrest in MDA-MB-231 cells (Yuan et al., 2018). Herein, both the induction of autophagy and S-phase arrest were first confirmed in MDA-MB-231 cells following the exposure to the combined regimen of 10 μM As^III^+6.4 μM Tetra for 48 h (**Supplementary Figures 1** and **2**). Previous studies have demonstrated a close association between autophagy and apoptosis as well as cell cycle arrest induction in different types of cancer cells including MDA-MB-231 (Cheng et al., 2015; Gao et al., 2015; Lee et al., 2016; Lin et al., 2016; Chen et al., 2019). In order to clarify whether there was a link between autophagy and apoptosis/necrosis as well as S-phase arrest, two autophagy inhibitors, 3-MA and wortmannin, were used in the current study. After treatment for 48 h with 10 μM As^III^+6.4 μM Tetra in the presence or absence of 3-MA (0.25 and 1.0 mM) or wortmannin (0.25 and 1.0 μM), the effects of inhibitors on the alteration of apoptosis/necrosis induction and cell cycle profiling in MDA-MB-231 cells were investigated. As shown in **Figure 4**, in comparison to the combined regimen-treatment group, the addition of 3-MA or wortmannin, regardless of the concentrations of each respective inhibitor, had little effect on the apoptosis/necrosis induction, indicating almost no association between autophagy and apoptosis/necrosis induction. It is worthy of note that the combined regimen-triggered S-phase arrest was successfully corrected by the addition of a relatively high concentration of 3-MA (1 mM) and wortmannin (1 μM), respectively (**Figures 5A, C**). Interestingly, a clear increase in the cell populations in the G_2_/M phase was concomitantly observed (**Figures 5A, B, D**).

### Involvement of JNK Activation in the Cytotoxicity of As^III^ Combined With Tetra in MDA-MB-231 Cells

**Figure 6:**
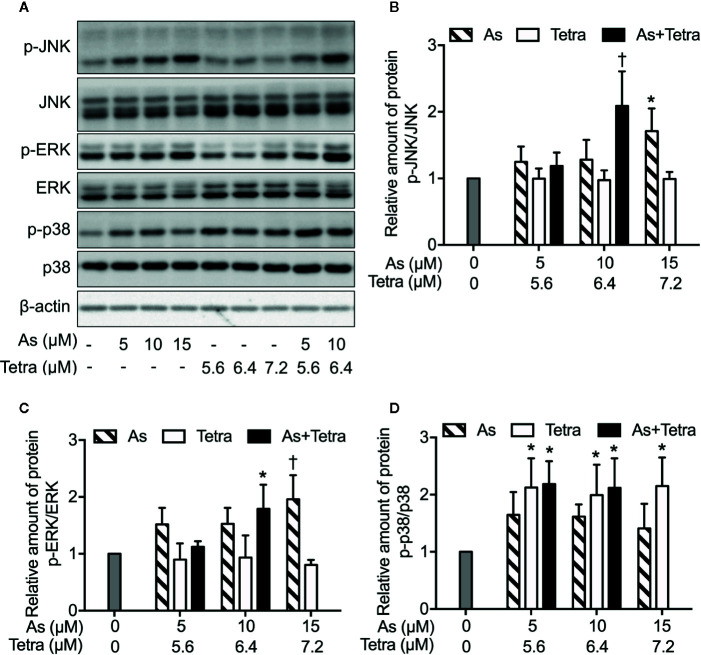
MAPK activation in MDA-MB-231 cells treated with As^III^ and Tetra, alone or in combination. **(A–D)** Following treatment for 48 h with the indicated concentrations of As^III^ and Tetra, alone or in combination, the expression profiles of both phosphorylated and total forms of JNK, ERK, and p38 were analyzed using western blotting. Representative images of the expression profile of each protein are shown from three independent experiments. The relative expression levels were expressed as the ratios between the phosphorylated active form and total form of each target protein expression levels, and compared with those of untreated control group, respectively. Results are shown as the means ± SD from three independent experiments. p-JNK, p-ERK, and p-p38 represent phospho-JNK, phospho-ERK, and phospho-p38, the respective phosphorylated active form of each MAPK. *p<0.05, ^†^p<0.01, vs. control. As, As^III^; Tetra, tetrandrine. Since enough cells cannot be collected in the group treated with 15 μM As^III^ in combination with 7.2 μM Tetra due to its strong cytotoxicity, western blot analyses were not conducted.

To explore whether MAPK signaling pathways are involved in the cytocidal effect of As^III^ combined with Tetra, the activation of JNK, p38 and ERK was determined in MDA-MB-231 cells following the treatment with the indicated concentrations of As^III^ and Tetra, alone or in combination, for 48 h. In comparison to control groups, exposure to As^III^ or Tetra exhibited little effect on the ratio of phospho-JNK/JNK, except for the highest concentrations of As^III^ (15 μM) with the capability to increase the ratio (**Figures 6A, B**). Of note, a substantial increase in the ratio of phospho-JNK/JNK was detected following the treatment with 10 μM As^III^ combined with 6.4 μM Tetra (**Figures 6A, B**). Similar alterations in the ratio of phospho-ERK/ERK were also detected (**Figures 6A, C**). As shown in **Figures 6A, D**, a significant increase in the ratio of phospho-p38/p38 was detected following the exposure to various concentrations of Tetra alone (5.6, 6.4, and 7.2 μM), and the increase was not influenced by the addition of As^III^. In addition, only a modest increase in the ratio of phospho-p38/p38 was observed following the exposure of As^III^ alone (5, 10, and 15 μM). These results indicated the activation of each MAPK in the cells treated with the combined regimen, although the degree of their activation was different to some extent.

**Figure 7:**
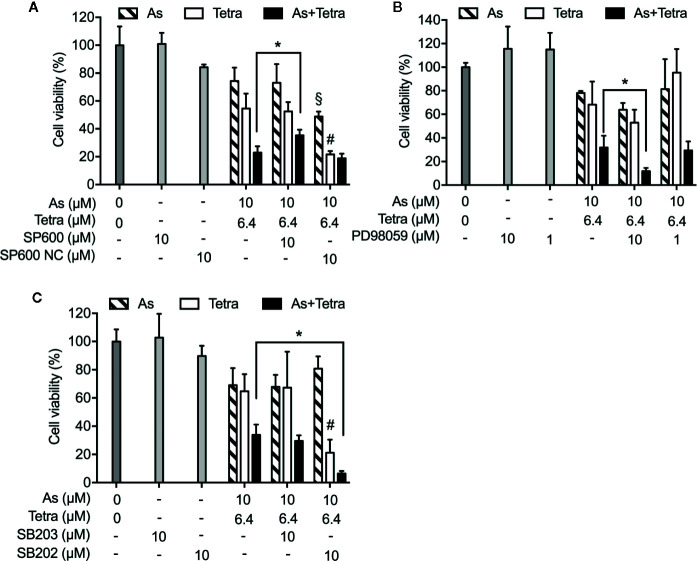
Abrogation of the cytotoxicity of As^III^ combined with Tetra by JNK inhibition in MDA-MB-231 cells. Following the treatment for 48 h with 10 μM As^III^ and 6.4 μM Tetra, alone or in combination, in the presence or absence of MAPK inhibitors and their negative controls [JNK inhibitor SP600125 and its negative control SP600125NC **(A)**; ERK inhibitor PD98059 **(B)**; p38 MAPK inhibitor SB203580 and its negative control SB202474 **(C)**], the cell viability of MDA-MB-231 was determined by XTT assay. Relative cell viability was calculated as the ratio of the absorbance at 450 nm of each treatment group against those of the corresponding untreated control group. Data are shown as the means and SD from more than three independent experiments. *p<0.01 vs. the combination; ^§^p<0.01, vs. 10 μM As^III^ alone; ^#^p<0.01, vs. 6.4 μM Tetra alone. As, As^III^; Tetra, tetrandrine; SP600, SP600125; SP600 NC, SP600125NC; SB203, SB203580; SB202, SB202474.

Next, in order to clarify whether the respective activation of JNK, p38 and ERK is implicated in the cytotoxicity, cell viability of MDA-MB-231 cells was investigated following the exposure for 48 h to 10 μM As^III^ and 6.4 μM Tetra, alone or in combination, in the presence or absence of potent inhibitors of JNK, p38, and ERK, respectively. Consistent with the results in **Figure 1**, a significant decrease in cell viability was induced by 10 μM As^III^ and 6.4 μM Tetra, each alone, and further strengthened by their combination (**Figure 7**). Notably, the combined regimen-triggered cytotoxicity was partially but significantly abrogated by the addition of 10 μM SP600125, an inhibitor for JNK, but not SP600125NC, a negative control for SP600125 (**Figure 7A**). Conversely, the addition of SP600125NC intensified the cytotoxicity of As^III^ and Tetra, each alone (**Figure 7A**). As shown in **Figure 7B**, the cytotoxicity of the combined regimen was hardly altered by 1 μM of PD98059, an inhibitor for ERK, however, was significantly augmented by 10 μM of PD98059. In addition, no alteration in the cytotoxicity of the combined regimen was observed regardless of the presence of 10 μM of SB203580, a specific inhibitor of p38 MAPK, indicating almost no involvement of p38 MAPK in the cytotoxicity (**Figure 7C**). The addition of 10 μM of SB202474, a negative control for SB203580, interestingly enhanced the cytotoxicity of 6.4 μM Tetra alone as well as the combined regimen, although there was no obvious plausible explanation for the enhancement right now (**Figure 7C**). All of MAPK inhibitors and their respective negative control itself had no effect on the cell viability of MDA-MB-231 (**Figure 7**).

### Implication of JNK Activation in the Cytotoxicity of MDA-MB-231 Cells Treated With As^III^ in Combination With Tetra Through Modulating Cell Cycle Progression

**Figure 8:**
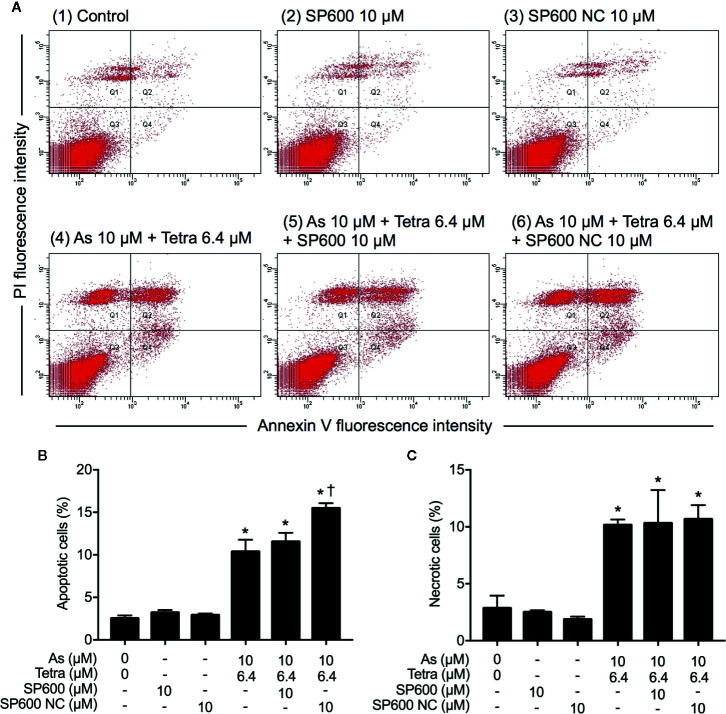
Effect of SP600125 on the induction of apoptosis and necrosis in MDA-MB-231 cells treated with As^III^ combined with Tetra. After treatment with 10 μM As^III^+6.4 μM Tetra in the presence or absence of 10 μM of SP600125 and its negative control SP600125NC for 48 h, cells were stained with annexin V-FITC and PI, and analyzed by flow cytometry. **(A)** Representative dot plots from three independent experiments are shown. The percentages of apoptotic cells **(B)** and necrotic cells **(C)** were quantified by the same manner as described in the legend of Figures 2 and 3. *p<0.0001 vs. control, ^†^p<0.0001, vs. 10 μM As^III^+6.4 μM Tetra. As, As^III^; Tetra, tetrandrine; SP600, SP600125; SP600 NC, SP600125NC.

**Figure 9:**
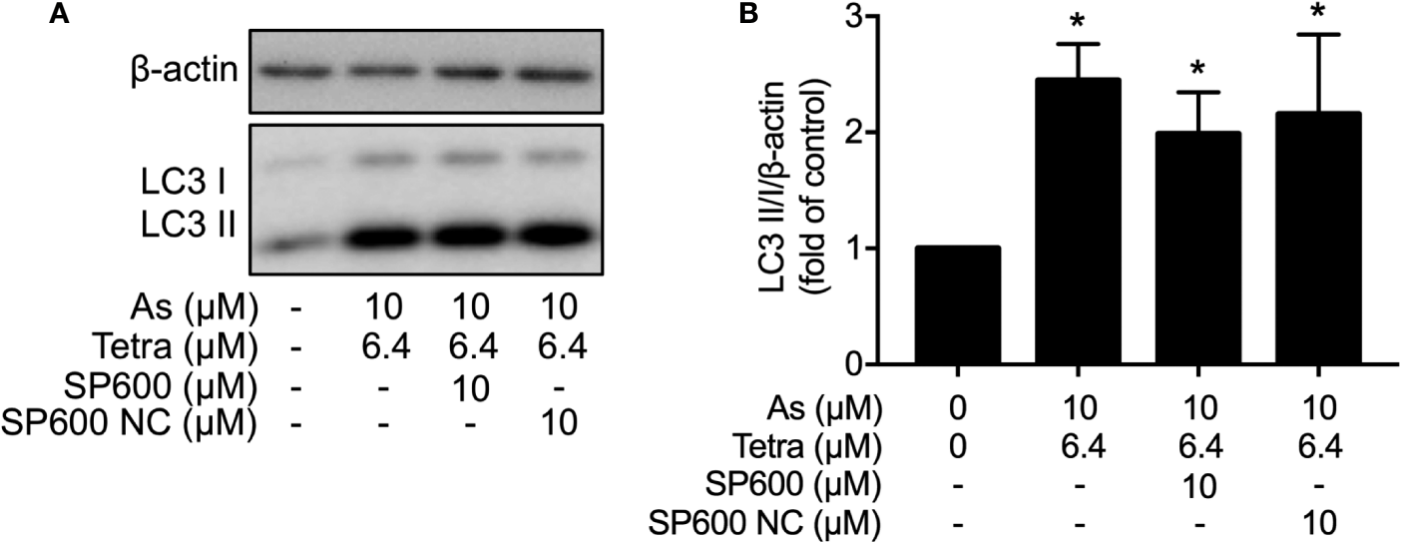
Effect of SP600125 on the induction of autophagy in MDA-MB-231 cells treated with As^III^ combined with Tetra. After treatment with 10 μM As^III^+6.4 μM Tetra in the presence or absence of 10 μM of SP600125 and its negative control SP600125NC for 48 h, the expression of LC3 protein was analyzed using western blot. **(A)** Representative images of the expression profile of LC3 are shown from three independent experiments. **(B)** The relative expression level was expressed as the ratio between LC3 protein and β-actin protein expression levels, and compared with those of untreated control group. Results are shown as the means ± SD from three independent experiments. *p<0.05 vs. control. As, As^III^; Tetra, tetrandrine; SP600, SP600125; SP600 NC, SP600125NC.

In order to provide detailed evidence for the implication of JNK activation in the combined regimen-triggered cytotoxicity, alterations of the induction of apoptosis and necrosis were first investigated following the exposure of MDA-MB-231 cells to 10 μM As^III^ combined with 6.4 μM Tetra in the presence or absence of SP600125 or its negative control for 48 h. As shown in **Figures 8A, B**, a significant increase in the number of apoptotic cells was not altered by the addition of SP600125, whereas the increase was slightly but significantly enhanced by the addition of SP600125NC. The combined regimen-triggered necrosis was also not affected by both SP600125 and its negative control, SP600125NC (**Figures 8A, C**), indicating little involvement of JNK activation in the induction of apoptosis and necrosis. In addition, substantial upregulation of the expression of LC3 was not affected by either SP600125 or SP600125NC (**Figure 9**), indicating that JNK activation and the induction of autophagy independently occurred in the cells.

**Figure 10:**
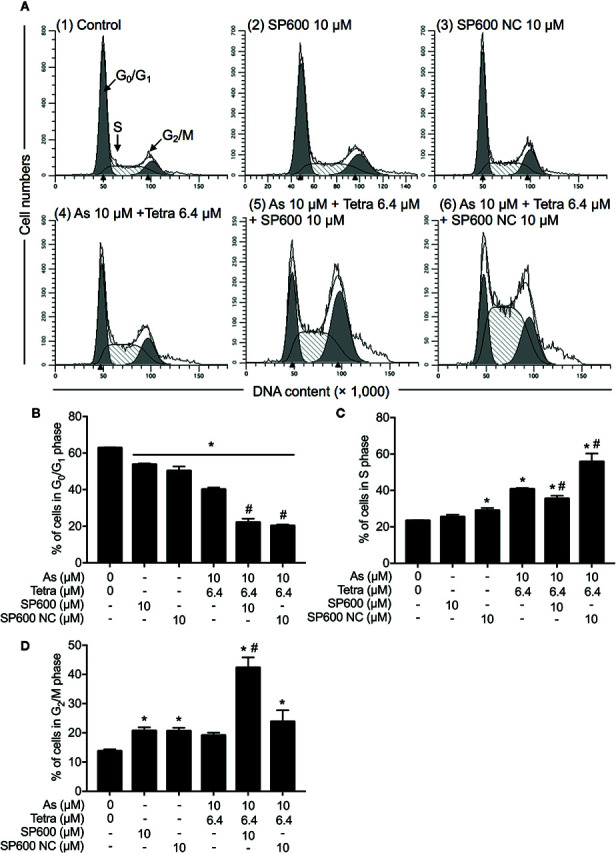
Association of JNK activation with cell cycle progression of MDA-MB-231 cells treated with As^III^ in combination with Tetra. **(A–D)** After treatment with 10 μM As^III^+6.4 μM Tetra in the presence or absence of 10 μM of SP600125 or its negative control SP600125NC for 48 h, cell cycle profiling was performed by FACSCanto flow cytometer. Analyzed data and profiles for each G_0_/G_1_ and G_2_/M phase using Diva software and ModFit LT™ ver.3.0. are shown in the gray area. Cells at S phase are shown as shaded area. A representative FACS histogram from three separate experiments is shown **(A)**. *p<0.05, vs. control; ^#^p<0.05, vs. 10 μM As^III^+Tetra 6.4 μM. As, As^III^; Tetra, tetrandrine; SP600, SP600125; SP600 NC, SP600125NC.

Next, the effect of SP600125 on the alteration of cell cycle profiling was further investigated. Consistent with results in **Figure 5** and **Supplementary Figure 2**, S-phase arrest along with a significant decrease in the cell populations in the G_0_/G_1_ phase was confirmed in MDA-MB-231 cells after treatment with 10 μM As^III^ combined with 6.4 μM Tetra for 48 h (**Figures 10A–C**). In comparison, S-phase arrest was modestly but significantly reversed by the addition of 10 μM SP600125 (**Figures 10A, C**). Of note, a remarkable increase in the cell populations in the G_2_/M phase along with a further decrease in the cell populations in the G_0_/G_1_ phase was concomitantly observed (**Figures 10A, B, D**). On the other hand, S-phase arrest was further enhanced by the addition of 10 μM SP600125NC (**Figures 10A, C**). Intriguingly, no alteration in the cell populations in the G_2_/M phase was observed when combining 10 μM SP600125NC to the combinatorial treatment, although a further decrease in the cell populations in the G_0_/G_1_ phase was recognized (**Figures 10A, B, D**). The addition of SP600125NC itself, but not SP600125, slightly but significantly induced S-phase arrest of the cells, although both of them induced measurable decrease and increase in the cell populations in the G_0_/G_1_ and G_2_/M phase, respectively (**Figures 10A, B, D**).

## Discussion

In this study, we demonstrated that the combined regimen of As^III^ and Tetra exerted a synergistic cytotoxic effect against T47D (**Figure 1**), which was in good agreement with our previous study on MCF-7 cells (Yao et al., 2017). In line with our recent work (Yuan et al., 2018), we also confirmed the synergistic cytocidal effect of the two drugs in MDA-MB-231 cells, and further indicated that MDA-MB-231 cells were markedly more susceptible to the combinatorial treatment than T47D cells (**Figure 1**). Collectively, As^III^ and Tetra, which have been used as medicinal agents, should be valuable in the development of novel therapeutic approaches to combat different types of breast cancers in spite of their estrogen dependency.

Despite the fact that the aim of anticancer therapy has been commonly focused on the induction of apoptosis in premalignant and malignant cells, the role of necrotic cell death in chemotherapeutic treatment has been increasing appreciated since tumor cells evolve diverse strategies to evade apoptosis during tumor development (Cui et al., 2011; Xu et al., 2014). In this regard, the combined regimen of a relatively low concentration of each drug (10 μM As^III^+6.4 μM Tetra) induced both apoptosis and necrosis in MDA-MB-231 cells (**Figure 2**). Intriguingly, the combined regimen of 15 μM As^III^+7.2 μM Tetra and TMA exhibited very similar apoptosis-inducing activity in MDA-MB-231 cells, providing very meaningful *in vitro* experimental data for breast cancer drug development, although more detailed analyses including *in vivo* experiments are obviously needed.

In addition to the induction of apoptotic/necrotic cell death, our results also demonstrated that autophagic cell death and S-phase arrest (**Supplementary Figures 1** and **2**) contributed to the cytocidal effects of the combined regimen of 10 μM As^III^+6.4 μM Tetra in MDA-MB-231 cells. Although autophagy has been linked to apoptotic/necrotic cell death in many cases (Nikoletopoulou et al., 2013; Yoshida, 2017; Chen et al., 2019), our experimental results demonstrated that the addition of either 3-MA or wortmannin, two autophagy inhibitors, successfully corrected the combined regimen-triggered S-phase arrest, however, had little effect on the apoptosis/necrosis induction (**Figures 4** and **5**). Autophagy has been demonstrated to act either as a cytoprotective process or a pro-death factor in different cellular contexts (White and DiPaola, 2009; Mathiassen et al., 2017). A recent review article has demonstrated that in response to different exogenous cellular stress stimuli, adequate autophagy can be activated to induce degradation of cell cycle arrest-related proteins such as p27, consequently contributes to tumorigenesis and/or drug resistance (Zheng et al., 2019). On the other hand, anticancer agents cause overactivated autophagy to breakdown cell cycle regulators including cyclin-dependent kinases or cyclin to induce permanent cell cycle arrest and autophagy-related cell death (Maes et al., 2013; Zheng et al., 2019). While the correlative induction of cell-cycle arrest and autophagy has been discussed, the molecular mechanisms linking them together remain poorly characterized (Maes et al., 2013; Mathiassen et al., 2017; Zheng et al., 2019). Based on the previous observations and our findings, we suggest that the abrogation of the overactivated autophagy might promote cell cycle progression, and consequently inhibit the cytotoxic effects of As^III^ combined with Tetra in MDA-MB-231 cells, although more detailed mechanisms underlying the crosstalk between cell cycle progression and autophagy are obviously needed to clarify.

We also demonstrated that the cytocidal effect of As^III^ combined with Tetra was significantly abolished by SP600125, a potent inhibitor of JNK, but not by SB203580, a specific inhibitor for p38, suggesting the contribution of JNK, instead of p38, to the cytotoxicity (**Figure 7**). In line with previous reports showing that ERK usually served as a survival mediator implicated in cytoprotection (Yao et al., 2012; Kikuchi et al., 2013), the combined regimen-triggered cytotoxicity was clearly augmented by PD98059, an inhibitor of ERK, suggesting that activation of ERK might compensate for the cytocidal stimuli. Although the activation of JNK has been deeply implicated in apoptosis/necrosis induction in different types of cancer cells (Deng et al., 2018; Qiao et al., 2019), inhibition of JNK by SP600125 did not alter the induction of apoptosis/necrosis (**Figure 8**), suggesting little involvement of JNK activation. Similarly, substantial upregulation of the expression of LC3 was not affected by SP600125 (**Figure 9**), suggesting that JNK activation and autophagy independently contributed to the cytotoxicity of As^III^ combined with Tetra in MDA-MB-231 cells.

In comparison, SP600125 modestly but significantly corrected S-phase arrest, which was accompanied by a significant increase and decrease in the cell populations in the G_2_/M and G_0_/G_1_ phase, respectively (**Figure 10**). These results thus suggested that the combined regimen-triggered cytotoxicity was attributed to JNK activation-associated with S-phase arrest. In agreement with this opinion, we interestingly observed that SP600125NC, a negative control for SP600125, significantly enhanced the cytotoxicity of As^III^ and Tetra, alone or in combination (**Figures 7** and **8**), which might be explained by the capability of SP600125NC to strengthen S-phase arrest (**Figure 10**) although the mechanisms underlying the strengthening remain to be clarified. Recently, Xie et al. demonstrated that Pu-erh tea water extract induced growth inhibition of MDA-MB-231 cells through S-phase arrest associated with upregulation of p21 and downregulation of cyclin D1, cyclin E, all of which was mediated *via* JNK activation (Xie et al., 2017). They further showed that co-treatment with SP600125 restored the water extract-induced alterations of p21, cyclin D1, and cyclin E, suggesting that S-phase arrest occurred through the activation of JNK-related cell death pathway (Xie et al., 2017). Most recently, Kong et al. have demonstrated that cardamonin, a naturally occurring chalcone isolated from large black cardamom, induces G_2_/M arrest and apoptosis in breast cancer cells including MDA-MB-231 (Kong et al., 2020). They further clarified that SP600125 blocked FOXO3a expression and nuclear translocation, and significantly diminished the expression of FOXO3a and the upregulation of p21 and p27, two target genes of FOXO3a (Kong et al., 2020). Similar to these previous reports, we recently also demonstrated that S-phase arrest associated with the upregulation of FOXO3a, p21, p27 along with decreased cyclin D1 expression contributed to the anticancer activity of As^III^ and Tetra against MDA-MB-231 cells (Yuan et al., 2018). Collectively, we suggest that the activation of JNK-FOXO3a pathway probably plays a critical role in the combined regimen-triggered cytotoxicity in MDA-MB-231 cells, although more detailed analyses are needed to clarify this opinion.

## Conclusion

Our results demonstrated that besides apoptosis/necrosis, autophagic cell death and cell cycle arrest were also involved in the cytotoxicity of As^III^ combined with Tetra in breast cancer cells, and that MDA-MB-231 cells were markedly more susceptible to the combinatorial treatment than T47D cells. Therefore, we suggest that the combined regimen could be a broadly applicable approach to combat different types of breast cancer cells. We further demonstrated that the activation of JNK and autophagy independently contributed to the cytotoxicity of As^III^ combined with Tetra *via* modulating cell cycle progression in MDA-MB-231 cells. TNBC has been characterized by highly aggressive metastatic behavior and represents one of the most difficult subtypes of breast cancer (Carey et al., 2010). In view of this, the combination of As^III^ and Tetra, both of which have been used in the clinic, should be valuable in developing a novel therapeutic strategy to cease the uncontrolled proliferation of cancer cells in patients with TNBC. Our findings provide valuable insights into the development of the novel therapeutic for different types of breast cancer, especially TNBC.

## Data Availability Statement

All datasets presented in this study are included in the article/**Supplementary Material**.

## Author Contributions

BYu and BYua contributed equally to this study. BYua conceived and designed the study and drafted the manuscript. BYu and BYua performed the experiments. JL, AK, HK, HH, XH, MO, MS, TH, YF, XP, and NT assisted interpretation of the results with BYua. All authors contributed to the article and approved the submitted version.

## Funding

This work was partially supported by The Japan Society for the Promotion of Science (JSPS) KAKENHI Grant to BYua (Grant Numbers 26460233) (Grant Numbers 17K08465). This work was also partially supported by National Natural Science Foundation of China (NSFC) to XP (Grant Numbers 81774319) (Grant Numbers 81560775). The funders had no role in the design of the study; in the collection, analyses, or interpretation of data; in the writing of the manuscript, or in the decision to publish the results.

## Conflict of Interest

The authors declare that the research was conducted in the absence of any commercial or financial relationships that could be construed as a potential conflict of interest.
