# Supplementary material for: JNK and Autophagy Independently Contributed to Cytotoxicity of Arsenite combined With Tetrandrine via Modulating Cell Cycle Progression in Human Breast Cancer Cells
Source: Front Pharmacol. 2020 Jul 17;11:1087. doi: 10.3389/fphar.2020.01087 (PMC7379898; doi:10.3389/fphar.2020.01087)
Supplement: Supplementary file 1 [file DataSheet_1.docx]

Supplementary Material

# Supplementary Data

Supplementary Material should be uploaded separately on submission. Please include any supplementary data, figures and/or tables. All supplementary files are deposited to FigShare for permanent storage and receive a DOI.

Supplementary material is not typeset so please ensure that all information is clearly presented, the appropriate caption is included in the file and not in the manuscript, and that the style conforms to the rest of the article. To avoid discrepancies between the published article and the supplementary material, please do not add the title, author list, affiliations or correspondence in the supplementary files.

# Supplementary Figures and Tables

For more information on Supplementary Material and for details on the different file types accepted, please see [here](http://home.frontiersin.org/about/author-guidelines#SupplementaryMaterial). Figures, tables, and images will be published under a Creative Commons CC-BY licence and permission must be obtained for use of copyrighted material from other sources (including re-published/adapted/modified/partial figures and images from the internet). It is the responsibility of the authors to acquire the licenses, to follow any citation instructions requested by third-party rights holders, and cover any supplementary charges.

## Supplementary Figures


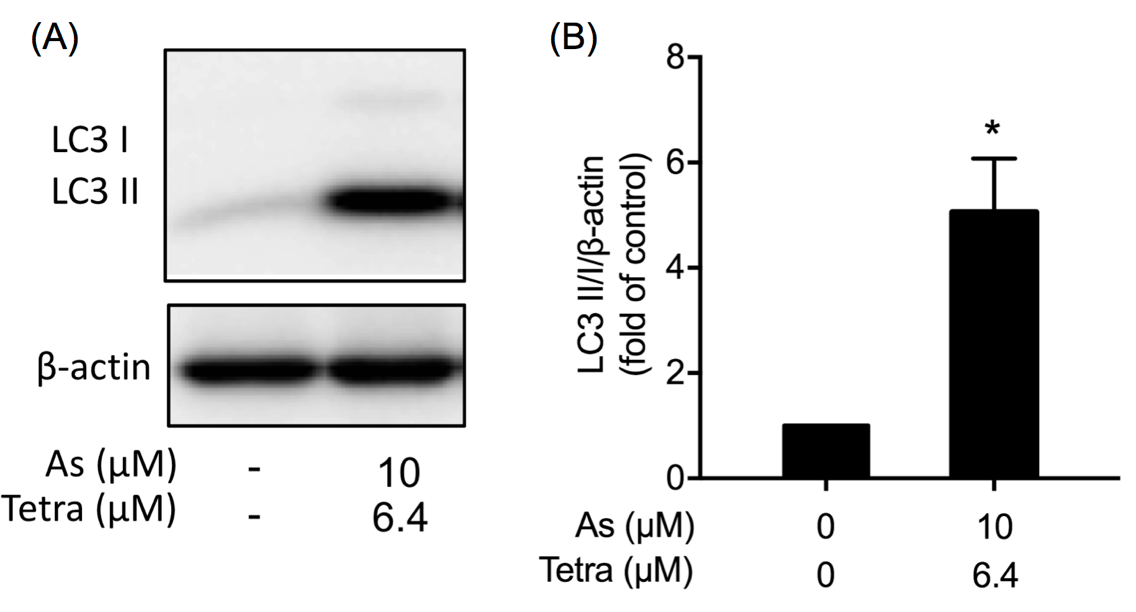


**Supplementary Figure 1.** Upregulation of LC3, an autophagic marker, in MDA-MB-231 cells treated with the combination of As^Ⅲ^ and Tetra. Following treatment with 10 μM As^III^ combined with 6.4 μM Tetra for 48 h, the expression of LC3 protein was analyzed using western blot as described in “Materials and methods”. (A) Representative image of the expression profile of LC3 is shown from three independent experiments. (B) The relative expression level was expressed as the ratio between LC3 protein and β-actin protein expression levels, and compared with those of untreated control group. Results are shown as the means ± SD from three independent experiments. *, p<0.01 vs. control. As, As^III^; Tetra, tetrandrine.


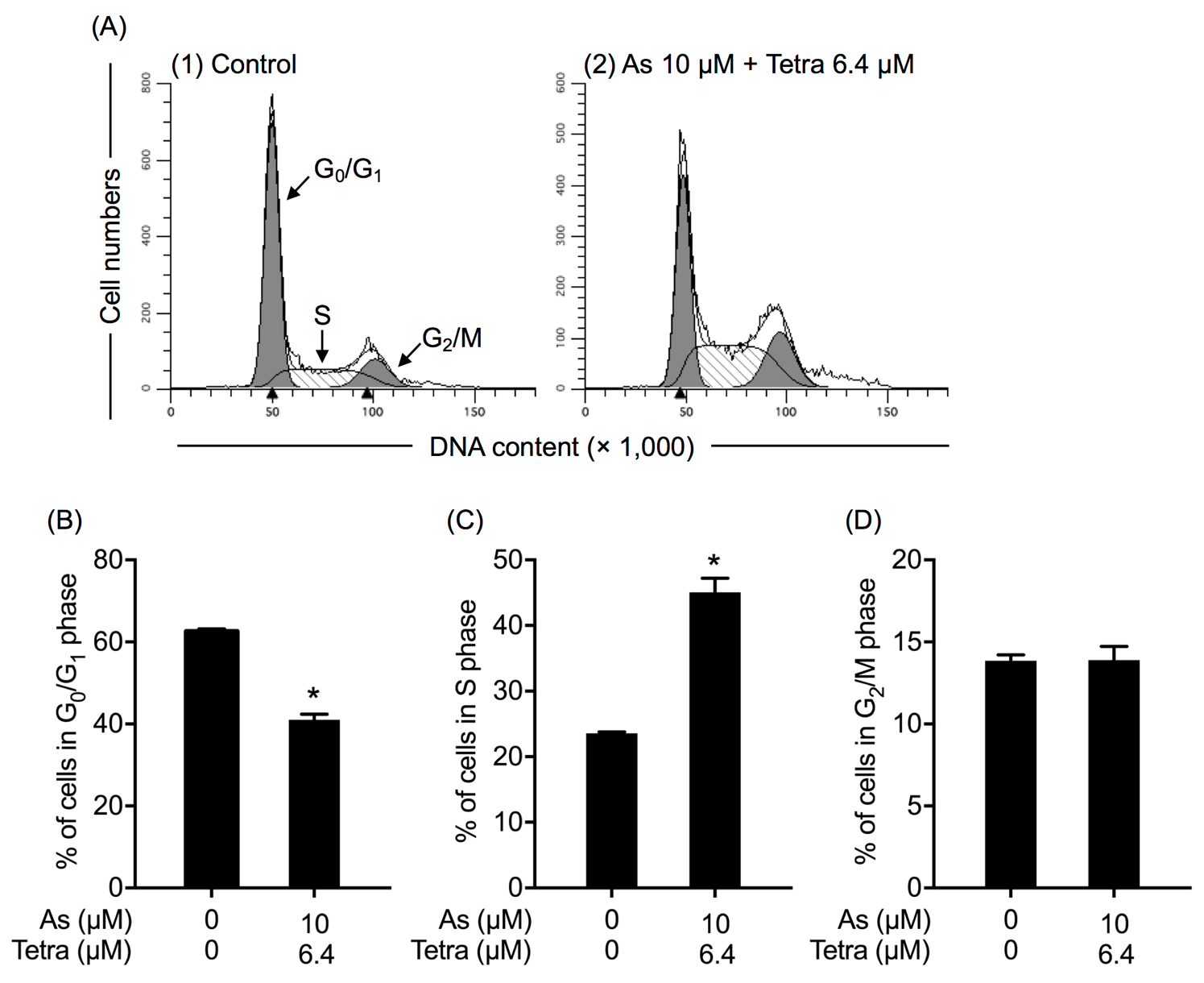


**Supplementary Figure 2.** S-phase arrest of MDA-MB-231 cells treated with As^Ⅲ^ combined with Tetra. (A-D) After treatment with 10 μM As^III^ combined with 6.4 μM Tetra for 48 h, cell cycle profiling was performed by FACSCanto flow cytometer as described under “Materials and methods”. Analyzed data and profiles for each G_0_/G_1_ and G_2_/M phase using Diva software and ModFit LT™ ver.3.0. are shown in the gray area. Cells at S phase are shown as shaded area. A representative FACS histogram from three separate experiments is shown (A). *, p<0.0001 vs. control. As, As^III^; Tetra, tetrandrine.
